# Do chewing gums and sweets containing xylitol prevent caries in children?

**DOI:** 10.1038/s41432-024-01018-2

**Published:** 2024-05-25

**Authors:** Darshini Ramasubbu, Brett Duane

**Affiliations:** 1 Dublin Dental University Hospital, Trinity College Dublin, Dublin, Republic of Ireland; 2 Trinity College Dublin, Dublin, Republic of Ireland

**Keywords:** Preventive dentistry, Dental public health

## Abstract

**Data sources:**

Three electronic databases (Pubmed, Embase and the Cochrane Library) were searched in December 2022, and again for additional literature on 3–5th January 2023. Reference lists of relevant systematic reviews were hand searched for other eligible studies for inclusion.

**Study selection:**

Randomised controlled clinical trials and controlled clinical trials conducted on children (aged ≤ 18 years), conducted between 1974–2022 and available in English, were eligible for inclusion. Studies were excluded if caries was not an outcome, the control group was not sufficient, they were lab-based studies or studies where xylitol delivery was not a sweet or chewing gum and where the xylitol product contained a component such as fluoride which may influence the outcomes.

**Data extraction and synthesis:**

Four calibrated reviewers independently screened titles and abstracts, and disagreements were resolved via group discussion. Preventative effect was determined by comparing the mean caries increment in the control and intervention groups, producing a preventative fraction. A total of 617 titles were initially screened for relevance. After duplicate removal, 268 abstracts were screened and 16 full text articles reviewed, with one more study then excluded. 10 studies investigated xylitol-containing chewing gum, and six looked at xylitol candy (one did both). Eight included studies were randomised controlled trials. Data extraction was undertaken by two reviewers.

**Results:**

3466 participants were included in the 10 studies that investigated xylitol chewing gum, and all 10 studies reported a statistically significant preventive effect compared to a no chewing gum or placebo control. In 9 studies, the preventive fraction was clinically significant. The six studies investigating xylitol candies contained a total of 1023 participants, and only one study demonstrated a significant preventative effect.

**Conclusions:**

There is some evidence that incorporating xylitol chewing gum daily has a caries-reducing effect in those with a moderate-to-high baseline caries level. This effect was not present for xylitol sweets.

## A Commentary on

**Pienihäkkinen K, Hietala-Lenkkeri A, Arpalahti I, Söderling E**.

The effect of xylitol chewing gums and candies on caries occurrence in children: a systematic review with special reference to caries level at study baseline. *Eur Arch Paediatr Dent* 2024; **25:** 145–160.

**GRADE Rating:** 

## Commentary

Xylitol is a naturally occurring sweetener, whose effect on dental caries is linked to increased saliva production, especially when used in a chewing gum^1^. It also likely decreases counts of bacteria associated with caries^1^. This topic area is important as it can identify products which may help prevent tooth decay, and provide guidance to dentists and allied professionals on the effectiveness of xylitol-containing products for their patients^2^.

The research questions were: ‘(1) can the consumption of xylitol chewing gum and/or candies reduce occurrence of dental caries in children, (2) what is the importance of the caries level of the children for the outcome, and (3) are the effects specific to xylitol?,’ and a systematic review is appropriate to answer these questions^2^. This review was registered in PROSPERO prior to data collection and it adhered to Preferred Reporting Items for Systematic Reviews and Meta-Analyses (PRISMA). PRISMA is an ‘evidence-based minimum set of items for reporting in systematic reviews and meta-analyses,’ which focuses on those that evaluate interventions^3^.

This systematic review aimed to evaluate randomised controlled clinical trials and controlled clinical trials published between 1974–2022 that investigated xylitol-containing chewing gums and sweets and their efficacy in preventing dental caries^2^. Whilst three electronic databases were searched more than once, and reference lists of relevant systematic reviews scrutinised for other eligible studies for inclusion, studies not available in English were excluded and there was no mention of contact with experts in the field or grey literature, which may limit the comprehensiveness of the search strategy^2^. Whilst previous systematic reviews related to this topic were identified in the introduction, it was not clear which or if all mentioned had their reference lists reviewed^2^. Clear eligibility criteria for study inclusion and exclusion were stated and only randomised controlled clinical trials and controlled clinical trials were included^2^. The Cochrane Risk of Bias tool was used to evaluate the included randomised controlled trials. A meta-analysis was not planned, due to expected heterogeneity and so descriptive analysis was instead used, alongside a table which summarised included studies, outlining their design, methods and results^2^. DMFS/dmfs or DMF/dmf were the primary outcome measures in the included studies, though some included enamel caries and used radiographs to aid detection^2^. Whilst this table stated the country where each study was undertaken, information on the socio-economic status of the participants would also have been useful^2^.

This systematic review concluded that incorporating xylitol chewing gum daily has a caries reducing effect, based on the statistically significant results of the 10 included studies which investigated this product, clinically significant preventative fractions in 9 included studies and other systematic reviews that have been conducted in this area^2^. However, only 5 of these studies were RCTs and there was variation in the proportion of xylitol in the delivered intervention, what the control group received, participant ages, and follow up periods, amongst other factors^2^. There is increased risk of bias in trials which do not use random allocation. Some included studies were double blinded, whereas others could not be as the control differed greatly from the intervention^2^. There was not a consistent caries reducing effect for the studies investigating the use of xylitol-containing sweets, with 5 RCTs included. It must be noted that there was variation between trials in the caries level of the included population and that baseline caries level is important when caries is an outcome^2^. Other limitations of the review include the quality and heterogeneity of the included studies, particularly the variety of the control measures, though the use of calibration in many included studies is a strength^2^. The authors of this review had anticipated this heterogeneity and additionally stated that all included studies were fair or low quality, with high or unclear risk of bias, which does effect the conclusions that can be drawn from this systematic review^2^.

This review focused on the paediatric population, those 18 years old and younger^2^. Study publication dates ranged from 1985 to 2015^2^. This topic would benefit from more randomised controlled trials, especially blinded ones, in patients with different levels of baseline dental caries, to investigate the applicability of the results to people at low risk of caries, alongside costs and adverse effects^2^. In terms of recommendations, this review suggests adding xylitol chewing gum to the daily diet of children at high or moderate caries level at baseline has a caries reducing effect, but that more research is needed on the type of gum and on xylitol sweets^2^. The authors highlight that xylitol is one component in caries prevention, and should be used in conjunction with dietary changes and fluoride toothpaste in those at risk of dental caries^2^.

Practice point

- There is limited evidence that the use of xylitol chewing gum prevents against caries in children in certain circumstances. It is important to focus preventive advice on dietary changes and the use of fluoridated toothpaste.

## References

[1] Nayak PA, Nayak UA, Khandelwal V (2014). The effect of xylitol on dental caries and oral flora. Clin Cosmetic Investig Dentistry.

[2] Pienihäkkinen K, Hietala-Lenkkeri A, Arpalahti I, Söderling E. The effect of xylitol chewing gums and candies on caries occurrence in children: a systematic review with special reference to caries level at study baseline. Eur Arch Paediatr Dent. 2024. 10.1007/s40368-024-00875-w. 10.1007/s40368-024-00875-w PMC11058973 38430364

[3] PRISMA. Home. 2024. Available from: http://www.prisma-statement.org/. Accessed 05/04/2024.

